# Spike Triggered Hormone Secretion in Vasopressin Cells; a Model Investigation of Mechanism and Heterogeneous Population Function

**DOI:** 10.1371/journal.pcbi.1003187

**Published:** 2013-08-15

**Authors:** Duncan J. MacGregor, Gareth Leng

**Affiliations:** Centre for Integrative Physiology, University of Edinburgh, Edinburgh, United Kingdom; University of Rochester, United States of America

## Abstract

Vasopressin neurons generate distinctive phasic patterned spike activity in response to elevated extracellular osmotic pressure. These spikes are generated in the cell body and are conducted down the axon to the axonal terminals where they trigger Ca^2+^ entry and subsequent exocytosis of hormone-containing vesicles and secretion of vasopressin. This mechanism is highly non-linear, subject to both frequency facilitation and fatigue, such that the rate of secretion depends on both the rate and patterning of the spike activity. Here we used computational modelling to investigate this relationship and how it shapes the overall response of the neuronal population. We generated a concise single compartment model of the secretion mechanism, fitted to experimentally observed profiles of facilitation and fatigue, and based on representations of the hypothesised underlying mechanisms. These mechanisms include spike broadening, Ca^2+^ channel inactivation, a Ca^2+^ sensitive K^+^ current, and releasable and reserve pools of vesicles. We coupled the secretion model to an existing integrate-and-fire based spiking model in order to study the secretion response to increasing synaptic input, and compared phasic and non-phasic spiking models to assess the functional value of the phasic spiking pattern. The secretory response of individual phasic cells is very non-linear, but the response of a heterogeneous population of phasic cells shows a much more linear response to increasing input, matching the linear response we observe experimentally, though in this respect, phasic cells have no apparent advantage over non-phasic cells. Another challenge for the cells is maintaining this linear response during chronic stimulation, and we show that the activity-dependent fatigue mechanism has a potentially useful function in helping to maintain secretion despite depletion of stores. Without this mechanism, secretion in response to a steady stimulus declines as the stored content declines.

## Author Summary

Vasopressin is a hormone that is secreted from specialised brain cells into the bloodstream; it acts at the kidneys to control water excretion, and thereby help to maintain a stable ‘osmotic pressure’. Specialised cells in the brain sense osmotic pressure, and generate electrical signals which the thousands of vasopressin neurons process and respond to by producing and secreting vasopressin. In response to these signals, vasopressin neurons generate complex “phasic” patterns of electrical activity, and this activity leads to vasopressin secretion in a complex way that depends on both the rate and pattern of this activity. We have now built a computational model that describes both how the vasopressin neurons generate electrical activity and also how that activity leads to secretion. The model, which gives a very close fit to experimental data, allows us to explore the adaptive advantages of particular features of the vasopressin neurons. This analysis reveals the importance of heterogeneity in the properties of vasopressin neurons, and shows how the vasopressin system is optimally designed to maintain a consistent hormonal output in conditions where its stores of releasable hormone are severely depleted.

## Introduction

Models of neuronal networks generally assume that the output of the neurons is well characterised by their spiking activity. However, for all neurons, their output is not the spikes themselves, but the neurotransmitter release that is triggered by those spikes, most commonly at synapses. Generally, the coupling between spike activity and transmitter release is nonlinear, subject to both frequency facilitation of release and to activity-dependent depression [1], and when, as is often the case, neurons fire spikes in complex patterns, these non-linearities mean that the spike activity itself can be a poor approximation of their true output. Detailed study at presynaptic terminals is difficult however, and the peptide secreting nerve terminals of the posterior pituitary have served as a more technically accessible model system [2], displaying similar complex activity patterning, and for which the characteristics of stimulus-secretion coupling have been well studied. Here we have studied the vasopressin neurons that project to the posterior pituitary, and develop a quantitatively precise model of both their spike activity and stimulus-secretion coupling, presenting a novel approach to modelling activity dependent facilitation and depression, based on abstractions of the underlying mechanisms.

The magnocellular neuroendocrine neurons of the hypothalamus synthesise and secrete the hormones oxytocin and vasopressin. These hormones can be readily measured in the bloodstream, have well-understood physiological roles, and are secreted subject to well-characterised reflex pathways. The rare ability to measure the electrical activity of identified neurons in physiological circumstances together with the resulting activity-dependent secretion [3] has meant that these neurons have become important model systems for the study of stimulus-secretion coupling in peptidergic neurons, and both the mechanisms leading to spike activity and those leading to secretion have been extensively studied by a wide range of experimental approaches.

By its actions at the kidney to regulate water loss, vasopressin has an essential role in the homeostatic regulation of plasma osmotic pressure. It is synthesised by magnocellular neurons in the supraoptic and paraventricular nuclei (SON and PVN) of the hypothalamus; these neurons project axons to the posterior pituitary gland, from where vasopressin is secreted into the blood stream following activity-dependent exocytosis of vesicles that are abundantly stored in axonal swellings and terminals. This secretion is triggered by spikes that are generated in the neuronal cell bodies and propagated down the axons. The vasopressin neurons are activated by increases in plasma osmotic pressure as a consequence partly of osmotically induced change in the cell volume, and partly of increased afferent input arising from other osmosensitive neurons in the forebrain [4], [5]. When activated, they display a distinctive “phasic” pattern of spike discharge, and it has been proposed that the adaptive advantage of this patterning is that it optimises the efficiency of stimulus-secretion coupling.

In the rat brain, there are about 9000 magnocellular vasopressin neurons, each of which individually generates spikes and manufactures and secretes hormone. These neurons are asynchronous in their spiking activity, and are quite heterogeneous, both in their levels of activity and in the membrane properties that determine specific features of their activity patterns. However, this individual phenotypic heterogeneity does not imply functional heterogeneity of the vasopressin cells: the physiologically important vasopressin signal is the plasma vasopressin concentration, which reflects the total secretion from the whole population.

Although individual vasopressin cells generate complex phasic patterns of electrical activity, the plasma vasopressin concentration increases remarkably linearly in response to increasing osmotic pressure above a “set point” [6], [7]. We have previously modelled the distinctive phasic spiking activity [8] in order to understand how this behaviour affects the coding of information in the vasopressin cell population. Because the phasic activity of vasopressin cells is asynchronous, and because of the properties of the mechanism that generates phasic activity, the average spike rate of the population increases relatively linearly in response to increased afferent input despite the short term non-linearity of individual cell responses.

However, at the nerve terminals, stimulus-secretion coupling is *also* highly non-linear, and how much hormone is secreted per spike depends on multiple features of the spike activity pattern. At the axonal terminals, vesicle exocytosis is triggered by spike-generated Ca^2+^ entry, and, in both vasopressin and oxytocin neurons, the spikes broaden as spike frequency increases, increasing the amount of Ca^2+^ entry [9], [10]. This spike broadening is thought to be partly responsible for an increase in secretion per spike that occurs with increasing frequencies of stimulation – a process called *frequency facilitation* [11]. This facilitation of vasopressin secretion peaks at about 15Hz, whereas oxytocin secretion per spike continues to increase up to frequencies of at least 50 Hz [12]. This difference suggests that in vasopressin terminals there is a competing, activity-dependent attenuation of stimulus-secretion coupling, possibly Ca^2+^-dependent inactivation of Ca^2+^ channels [13].

On a longer timescale, an additional mechanism produces activity-dependent *fatigue* of stimulus-secretion coupling in vasopressin cells (and to a much lesser extent in oxytocin cells) [14]. When axon terminals are stimulated at 13 Hz, the amount of vasopressin secreted declines progressively after about 10 s. However, if stimulation is interrupted by a silent period of 10 s or longer, the secretory response recovers. It was thus proposed that the function of phasic spiking is to optimise the secretion response, by minimising the consequences of fatigue while maximising those of facilitation [15]–[17].

However, there is a circularity in this logic. The differences between oxytocin and vasopressin neurons show that phasic firing is only efficient in vasopressin neurons because the particular properties of the vasopressin terminals make it so. We recently argued that there are *other* functional advantages to the phasic discharge pattern, and that the secretion mechanism in vasopressin cells may thus have evolved to confer efficiency on a spike pattern that has evolved for other reasons.

Our objective here was to develop a model of stimulus-secretion coupling in vasopressin cells that concisely reproduces facilitation and fatigue, and which quantitatively and qualitatively matches experimentally observed responses to stimulation. The broader aim is to test how these features relate to the ability of vasopressin neurons to respond to osmotic pressure. By coupling the secretion model to our spiking model we can simulate secretion response to varied input activity. One of the challenges is to understand how the highly heterogeneous and non-linear vasopressin neurons act together to produce a highly linear secretion response to increasing osmotic input. Is cell heterogeneity just unavoidable noise, or does it have a useful functional role?

Our combined model shows that cell heterogeneity, in the form of varied input intensity across the population, combined with phasic patterning, acts to linearise the population response, achieving a profile of secretion that matches that observed *in vivo*. Interestingly, the linearising effect of heterogeneity only works in combination with phasic spiking, and not on its own. Thus in vasopressin cells, the non-linear stimulus secretion properties of the nerve terminals, in conjunction with the pattern generating characteristics of the vasopressin cells, and in conjunction with heterogeneity in the vasopressin cell population, all combine to generate robust linear stimulus-secretion coupling across a wide dynamic range of input.

## Methods

### Modelling spike generation

The spike generating mechanism is modelled as described previously [8], using an integrate-and-fire based spiking model modified to include a set of activity-dependent effects on excitability [18] that shape spike patterning and produce emergent bistability. The modifications include a hyperpolarising afterpotential (HAP), a fast depolarising afterpotential (DAP), a slow afterhyperpolarisation (AHP), and a slow DAP based on a Ca^2+^-inactivated K^+^ leak current. The positive feedback of the activity-dependent slow DAP triggers bursting, but it is also opposed by the slower action of activity-dependent dendritic dynorphin release, which inactivates the DAP. The opposing effects combined with the random perturbations of the synaptic input produce a bistability that generates the successive periods of bursting and silence.

Setting the K^+^ leak (slow DAP) conductance (spiking model parameter *g_L_*) to zero removes the mechanism that underlies bistability, producing model cells with very similar interspike interval distributions to vasopressin neurons but which fire continuously rather than phasically. We use this here to compare otherwise identical phasic and non-phasic cells.

Synaptic input is simulated using a Poisson random process to generate EPSPs and IPSPs, represented by small (2 mV) exponentially decaying positive and negative perturbations to the membrane potential. The spike outputs match experimental observations very closely, and the parameters of the model can be fit to *in vivo* recorded data to produce indistinguishably close fits to multiple statistical measures of spike patterning. Importantly the model also reproduces the observed *in vivo* behaviour in response to an increasing rate of input activity; matching the shift from slow irregular spiking, to phasic spiking, and eventually to continuous spiking activity.

### Modelling stimulus-secretion coupling

Our aim was to concisely model the mechanisms of stimulus-secretion coupling in a way that gives a robust qualitative and quantitative fit to the characteristics of spike driven secretion as measured experimentally. As a major simplification, we model secretion from a single cell as from a single compartment, rather than from thousands of individual terminals. We assume that the summed effect of the stochastic, discrete, and relatively rare secretion events at individual terminals can be approximated by a continuous model. The large number of terminals (∼2000) is likely to make this a safe assumption. It may be interesting to make a more detailed model including individual terminals, but we do not have the detailed experimental data against which to fit such a model.

We fitted the model to experimental data reported in various studies of stimulus-secretion coupling *in vitro* in which vasopressin secretion from the isolated pituitary gland was measured in response to different patterns of electrical stimulation. These observations led to the description of stimulus-secretion coupling as being influenced by frequency facilitation, and by fatigue. Vesicle exocytosis depends on spike-evoked Ca^2+^ entry, and the rate of secretion depends on both the ion channels that propagate spikes and allow Ca^2+^ to enter, and the exocytotic apparatus that turns the Ca^2+^ signal into secretion. Frequency facilitation is thought to mainly reflect a modulation of Ca^2+^ entry in response to spike activity, using a spike broadening based mechanism detailed below. Modulation of the exocytotic response we include in the model by using a depletable vesicle store (see below).

Two hypotheses have been proposed to explain fatigue; that it is due to depletion of a readily-releasable pool of vesicles, or due to suppression of the secretion mechanism by a Ca^2+^-activated K^+^ conductance. The idea of depletion has previously been used to model secretion fatigue in oxytocin and vasopressin neurons [19], and in other types of hormone-secreting cells [20]–[22]. However, these models have been used to fit experimental data where cells have been depleted by large and prolonged depolarisation. In recent work [23] we used detailed quantitative data to predict the rate of vesicle release in response to spike stimulation during physiological conditions, showing that release events at individual axonal terminals are rare, requiring several hundred spikes to trigger a single release event. It seems unlikely therefore that fatigue on the timescale of seconds is due to depletion of vesicle stores. Thus, we have based our present model on the likely effects of a Ca^2+^-activated K^+^ mechanism analogous to the mechanism underlying the afterhyperpolarisation observed at vasopressin cell bodies [24].

### Spike conduction and secretion mechanisms

Spikes generated in the cell body are conducted down the axon to invade the secretory terminals – though whether a spike will invade a given terminal depends on the excitability of that terminal at the time of arrival of the spike. Like spikes at the cell body, spikes at the secretory terminals involve voltage-activated Na^+^ and Ca^2+^ channels, and terminal excitability (membrane potential) is modulated by various K^+^ conductances. Spike broadening is thought to be due to the voltage sensitive suppression of a K^+^ conductance, as demonstrated by experiments using the K^+^ channel blocker tetra-ethyl ammonia [2], [25].

**Figure 1:**
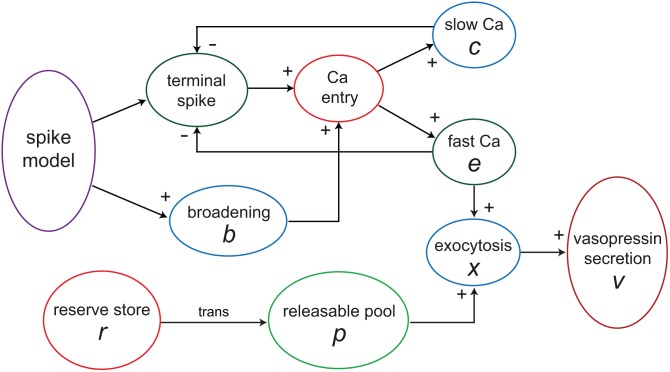
The vasopressin secretion model. Schematic illustrating the structure of the differential equation based single neuron secretion model. The model takes as input either a regular spike protocol or the output from the integrate-and-fire based spiking model. For a single vasopressin cell, secretion occurs from about 2,000 terminals and swellings; in the model secretion is represented as coming from a single compartment; thus secretion is treated as a single continuous variable rather than many discrete stochastic variables. In the model, Ca^2+^ entry is modulated by both fast (*e*) and slow (*c*) Ca^2+^ variables through their modulation of axonal terminal excitability, and is also a function of spike broadening (*b*). The secretion rate (vesicle exocytosis) is the product of the releasable pool (*p*) and the fast Ca^2+^ variable (*e*). When depleted, pool *p* is refilled from a reserve store (*r*) at a rate dependent on the store content.

**Figure 2:**
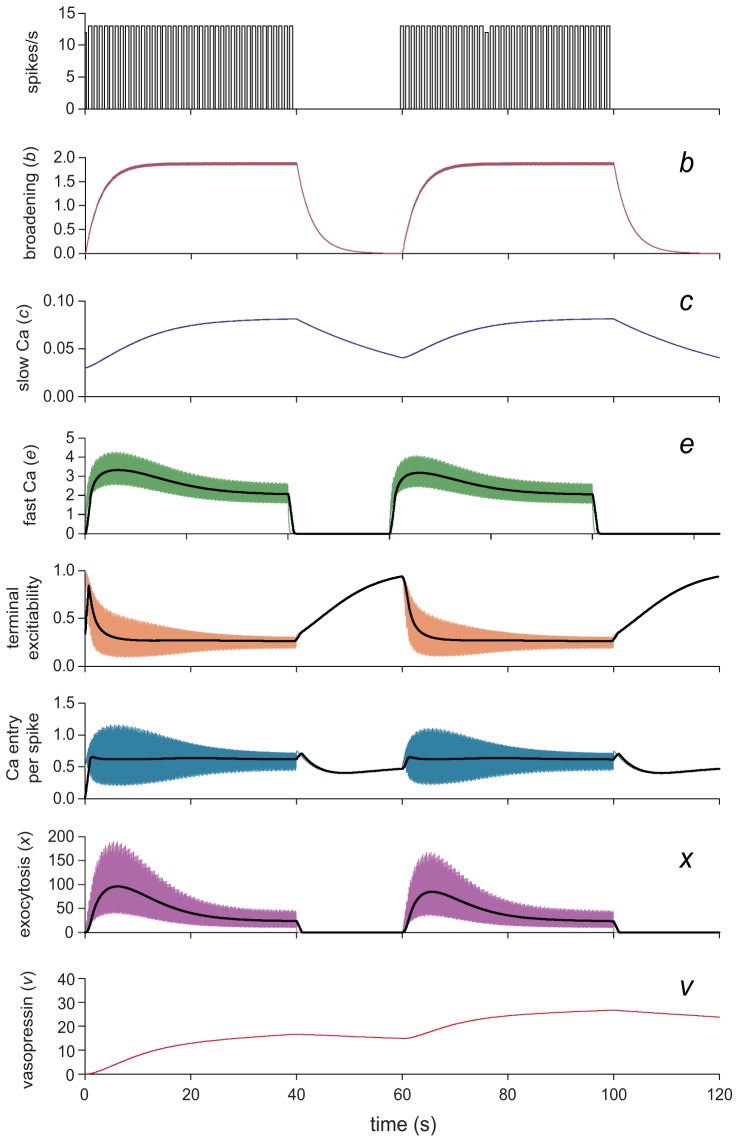
The secretion model's Ca^2+^ entry and vesicle release mechanisms. Here we simulate stimulation by two 40-s bursts of 13 Hz spikes at a regular 13 Hz, separated by 20 s. Each spike triggers a step increment of the exponentially decaying variables *b* (spike broadening), *c* and *e*. The step size for *c* and *e* depends on terminal excitability, which is modulated by two Hill equation functions of *c* and *e*, producing fast and slow negative feedback. Ca^2+^ entry per spike is a function of all three variables. The rate of exocytosis (secretion, *x*) is the scaled cube of *e*. Variable *v* accumulates *x* with a half-life of 2 min representing the resulting plasma vasopressin concentration. The competing effects of facilitation and the fast negative feedback combine to match the spike frequency secretion response. The slow negative feedback reproduces fatigue of stimulus-secretion coupling during prolonged stimulation. *x* shows an initial rise (facilitation) followed by a decline (fatigue) which recovers during the silent period as *c* decays restoring terminal excitability. The black lines show the data smoothed using a 1-s window.

The other effects on terminal excitability appear to depend on Ca^2+^ entry. Ca^2+^ acts at the secretory terminals on at least two different timescales. Vesicle exocytosis depends more on the rate of Ca^2+^ entry than on intracellular Ca^2+^ concentration, and it is likely that exocytosis occurs at sites that are close to clusters of voltage-gated Ca^2+^ channels. These submembrane sites experience high Ca^2+^ concentrations in response to spike activity, but only transiently, as the Ca^2+^ swiftly diffuses into the cytosol. This is represented in the model (Figure 1) by making the rate of secretion proportional to a ‘fast’ Ca^2+^ variable *e* that represents the sub-membrane Ca^2+^ concentration; spike broadening affects secretion by producing a larger rise in *e*. However, as mentioned above, the extent of facilitation of stimulus-secretion coupling in vasopressin terminals is limited by a competing, activity-dependent attenuation of stimulus-secretion coupling; we model this (Figure 2) as arising from Ca^2+^-dependent inactivation of Ca^2+^ channels [13].

Prolonged pulse stimulation of terminals results in an eventual increase in the rate of failure of spike propagation at the terminal [26], [27], and this is thought to be due to intracellular (cytosolic) Ca^2+^ accumulation [10] and the activation of a highly [Ca^2+^]_i_ sensitive, slow activating Ca^2+^-activated K^+^ conductance [28]. Thus in the model, a slow Ca^2+^ component *c* (reflecting cytosolic Ca^2+^ concentration) acts to reduce the terminal spike response. Its slow rate of accumulation and decay reproduces the slow development of fatigue, and the recovery during silent periods.

### Vesicle storage and the releasable pool

The model also includes a finite vesicle store so that we can assess the useful function of these mechanisms in maintaining long-term response. The store has two compartments: a large reserve store (*r*), and a smaller releasable pool (*p*) representing those vesicles in the axonal terminals that are relatively available for release.

### Model equations

The secretion model consists of a set of differential equations which take as input the spike events generated by the spiking model, or defined by a stimulation protocol.

The variables representing spike broadening (*b*), cytosolic Ca^2+^ concentration (*c*) and submembrane Ca^2+^ concentration (*e*) are all incremented with each spike. Broadening (*b*) is incremented by a fixed step *k_b_*, and decays exponentially with half-life λ*_b_*, converted to time constant τ*_b_*:
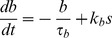
(1)where *s* = 1 if a spike is fired at time *t*, and *s* = 0 otherwise. All half-life parameters are converted to time constants using the formula in [8]. The Ca^2+^ variables, *c* and *e*, are incremented at a rate governed by Ca^2+^ entry (Ca_ent_), with similar exponential decay:
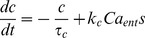
(2)

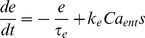
(3)Ca^2+^ entry depends on spike broadening (*b*), and is subject to submembrane and cytosolic Ca^2+^-dependent inactivation:
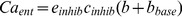
(4)where parameter *b_base_* gives a basal level for *b*. Entry is inhibited by *c* and *e* using two inverted Hill equations [29] with threshold and coefficient parameters, c*_θ_*, e*_θ_*, *c_n_* and *e_n_*:
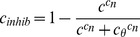
(5)

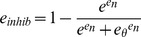
(6)The releasable vesicle pool (*p*) is depleted with secretion, *x*, and refilled at a rate proportional to the reserve store (*r*), unless already full (*p* = *p*_max_). The refill rate is scaled by parameter β:
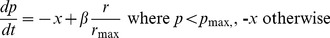
(7)The reserve store (*r*) is depleted exponentially as it refills *p*, with its maximum (and initial) value defined by parameter *r*_max_:
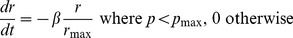
(8)The rate of secretion (vesicle exocytosis, *x*), is the product of the cube of the fast Ca^2+^ variable (*e*) and the releasable pool (*p*):
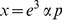
(9)We use a cube of *e* because Ca^2+^ activation of exocytosis is thought to be cooperative, and proportional to at least the square of the Ca^2+^ concentration close to the binding sites [30]; we found that using the cube gives a better fit to the *in vitro* data. Parameter α scales secretion to units that can be compared with experimental data. The final output of the model, vasopressin plasma concentration (*v*), increases with secretion (*x*) and decays with half-life λ*_v_*:
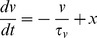
(10)The parameter values for the figures in this paper are given in Tables 1 and 2. The secretion model variables were initialised to 0, except *c* = 0.03, *p* = *p*_max_, and *r* = *r*_max_.

**Table 1:** Spiking model parameters.

Name	Description	Value	Units
*I*_re_	excitatory input rate	600	Hz
*I*_ratio_	inhibitory input ratio	1	-
*e*_h_	EPSP amplitude	2	mV
*i*_h_	IPSP amplitude	−2	mV
λ*_syn_*	PSP half-life	7.5	ms
*k_HAP_*	HAP amplitude per spike	60	mV
λ*_HAP_*	HAP half-life	9	ms
*k_DAP_*	fast DAP amplitude per spike	0.5	mV
λ*_DAP_*	fast DAP half-life	150	ms
*k_AHP_*	AHP activation factor	0.00012	mV/nM
λ*_AHP_*	AHP half-life	10000	ms
*C_AHP_*	minimum [Ca]_i_ to activate AHP	200	nM
*C*_rest_	rest [Ca]_i_	113	nM
*k_C_*	[Ca]_i_ increase per spike	11	nM
λ*_C_*	[Ca]_i_ half-life	2500	ms
*k_D_*	dynorphin activation per spike	2.693	a.u.
λ*_D_*	dynorphin half-life	7500	ms
*k_L_*	K^+^ leak (slow DAP) Ca^2+^ sensitivity	36	nM
*g_L_*	K^+^ leak maximum voltage	8.5	mV
*V*_rest_	resting potential	−56	mV
*V*_thresh_	spike threshold potential	−50	mV

**Table 2:** Secretion model parameters.

Name	Description	Value (Units)
*k_b_*	broadening per spike	0.05
λ*_b_*	broadening half-life	2000 (ms)
*b*_base_	basal spike broadening	0.5
*k_c_*	max cytosolic Ca^2+^ per spike	0.0003
λ*_c_*	cytosolic Ca^2+^ half-life	20000 (ms)
*k_e_*	max submembrane Ca^2+^ per spike	1.5
λ*_e_*	submembrane Ca^2+^ half-life	100 (ms)
*c_θ_*	threshold, terminal inhibition by *c*	0.07
*c_n_*	gradient, terminal inhibition by *c*	5
*e_θ_*	threshold, terminal inhibition by *e*	2.8
*e_n_*	gradient, terminal inhibition by *e*	5
β	pool refill rate scaling factor	50
*r*_max_	reserve store maximum	1000000 (pg)
*p*_max_	releasable pool maximum	5000 (pg)
α	secretion scaling factor	0.0005 (pg/unit)
λ*_v_*	plasma vasopressin half-life	120 (s)

### Implementation

The differential equations were integrated using the first order Euler method. We can do this safely since the step size (1 ms) inherited from the spiking model is much smaller than any of the time constants in the secretion model. Using the same fixed time step makes it simple to couple the secretion model to the integrate-and-fire based spike model.

The modelling software was developed in C++, using the open source wxWidgets graphical interface library. A typical run of the full model, simulating 2000s of activity for a population of 100 neurons takes ∼20s on an Intel i7-2600K quad core processor.

### Population heterogeneity

To generate a heterogeneous model cell population, we applied random variation to the rates of synaptic input received by each cell using the formula:
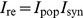
(11)where *I*_re_ is the spiking model's input rate parameter, *I*_pop_ is the population input rate, and *I*_syn_ is a randomly generated value using a lognormal distribution (see Results) with mean = 0 and standard deviation = 0.5, representing the synaptic connection density at a single neuron.

## Results

The model's Ca^2+^ entry and exocytosis mechanism was first tested by simulating stimulation with bursts of regularly spaced spikes and fixed lengths of bursts and silences. These protocols mimic the experimental protocols that were used in studies of vasopressin secretion from the isolated pituitary gland, the results of which were used to fit the model parameters [12], [14], [15], [31]. The most commonly used experimental protocol involved regular stimulation at 13 Hz for varying durations.

### Spike broadening and facilitation

When the vasopressin nerve terminals of the posterior pituitary are stimulated electrically with brief pulses that trigger spikes in the axons, the amount of vasopressin secretion depends on the stimulation frequency: the secretion per stimulus pulse (or per spike) increases to a maximum at ∼15 Hz, beyond which it gently declines again. Our hypothesis is that this rise and fall is due to two competing activity-dependent mechanisms.

At the beginning of a train of spikes, successive spikes broaden, and Ca^2+^ entry per spike increases. We fitted the spike broadening parameters, *k_b_*, *b*_base_, and λ*_b_* using the experimental data on spike broadening in [9] and [2]. A relatively slow decay is used, based on [31] which suggests that facilitation continues to increase over several seconds. The experimental data show a broadening of up to ∼100%, but this includes the Na^+^ current proportion of the spike, and so assuming that the broadening involves only the Ca^2+^ current (as Ca^2+^ removal suggests), the proportional increase in the Ca^2+^ proportion of the spike, which we model, is larger. Thus the parameters *b*_base_ and *k_b_* are fitted to give a value for the Ca^2+^ component (*b + b*_base_) ranging from 0.5 to ∼2.5, illustrated in the *b* trace of Figure 2.

**Figure 3:**
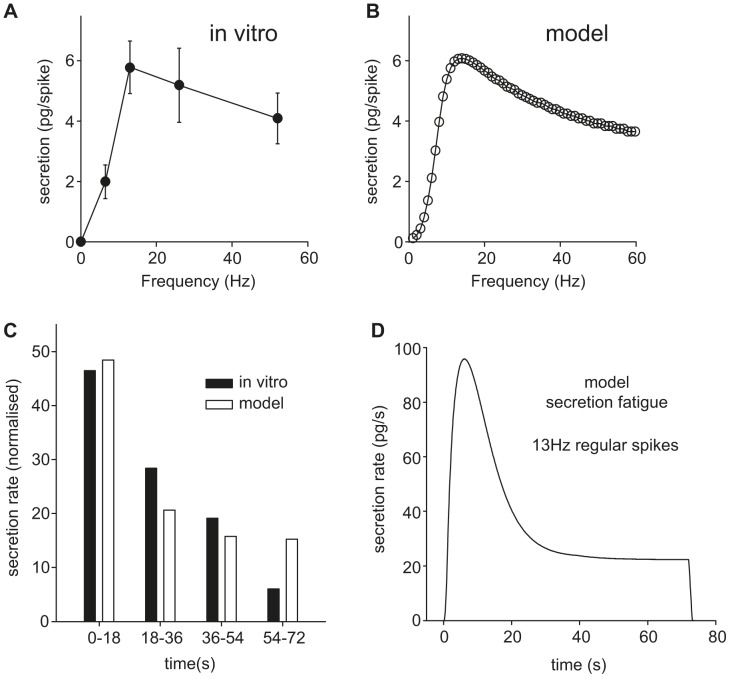
The secretion model fitted to experimental data. (**A**) Data (redrawn from [12]) shows vasopressin secretion per spike from a rat pituitary stimulated with a fixed number of stimulus pulses (156) at varied frequencies, producing a frequency response profile showing an initial climb to a peak at 13 Hz followed by a slower decline. (**B**) The same experiment reproduced in the model with frequency ranging from 1 Hz to 60 Hz in 1 Hz steps. The model combines frequency facilitation and competing fast negative feedback modulating Ca^2+^ entry to match the *in vitro* data. (**C**) Experimental data (redrawn from [14]) shows secretion rate measured in four consecutive 18-s periods during regular stimulation at 13 Hz, showing a progressive fatigue of the secretion response. The model tested with the same protocol shows a similar decline, though fails to match the last point. The experimental data come from a series of experiments in which glands were stimulated repeatedly for different durations with different orders of presentation. (**D**) The same model run as **C**, plotted to show a detailed temporal profile. This shows the initial facilitation of secretion, followed by a slow fatigue.

The competing inhibitory component (modelled as Ca^2+^-dependent inactivation of Ca^2+^ entry) uses both the fast and slow Ca^2+^ variables (*e* and *c*) to reduce Ca^2+^ entry per spike. The parameters for *e* and its inhibitory Hill equation were fitted to match the experimental data from [12] shown in Figure 3A. This frequency profile shows the sum of the effects of spike broadening and the competing inhibition, closely matched by the model in Figure 3B. These data were also used to adjust parameter α to scale the model's secretion units.

### Spike response and fatigue

*Fatigue* of the secretion response occurs over a timescale of tens of seconds, and recovery occurs on a similar timescale after stimulation is ended. The cytosolic Ca^2+^ concentration changes on a similar timescale, rising during stimulation, and decaying with a half-life of ∼20s [2]; our hypothesis for the model is that fatigue is due to the accumulation of cytosolic Ca^2+^, which activates a Ca^2+^-dependent K^+^ current that hyperpolarises the terminals, leading to an increased rate of spike failure and reduced Ca^2+^ entry.

The model parameters were fitted using the experimental data in [14]. We analysed the model secretion data by summing the secretion rate (*x*) values over each of four 18-s steps, to give a measure analogous to the cumulative secretion that was measured experimentally. We set λ*_c_*, the half-life for the cytosolic Ca^2+^ variable *c*, to 20s, and manually fit parameters *k_c_*, θ_c_ and *c*_n_ to match the fatigue profile in [14], as measured in response to stimulation with regular 13 Hz spikes (Figure 3c). The weakest fit of the model is to the 54–72 s period. This data point comes from a much lower n value than the others and so is less precisely determined, although margins of error are not provided because of the complex analytical approach that was used [14]. We thus consider that, as far as we can judge, the model is consistent with all available data, given the likely margins of error in those data. The detailed secretion rate profile is shown in Figure 3D.

### Secretion response – regular vs phasic stimulation

**Figure 4:**
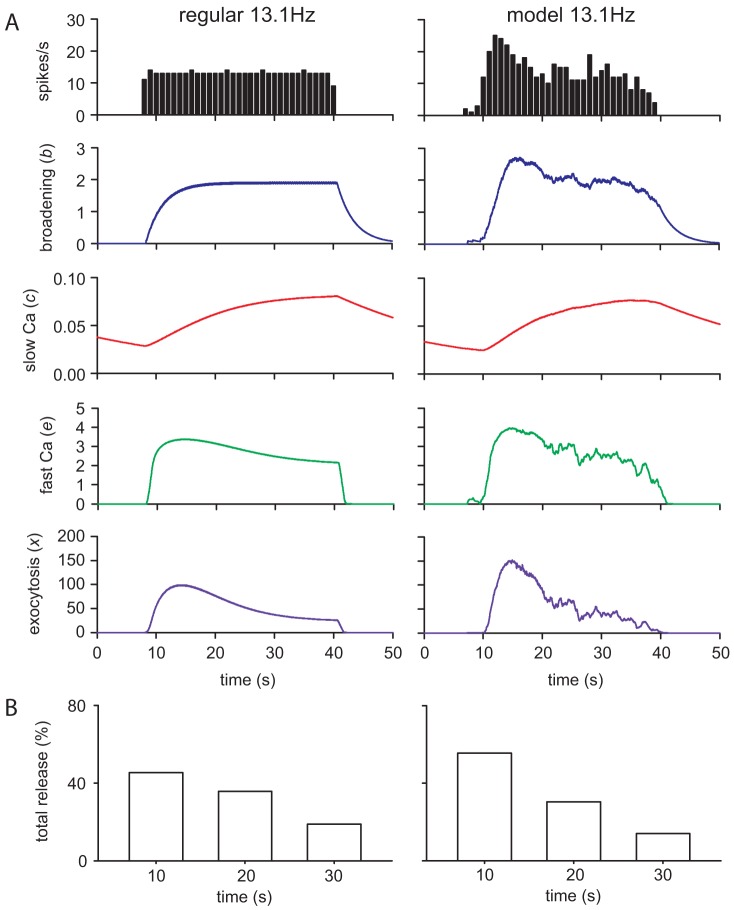
Secretion response comparing regular and burst profile stimulation. (**A**) Similar data to Figure 2 compares variables *b*, *e*, *c*, and the secretion rate x during regular (left) and burst (right) stimulation at a mean rate of 13.1 Hz and duration 32 s, comparable to the experimental data of [17]. The faster spiking at the head of the burst produces an increased initial rate of secretion, followed by a more rapid decline compared to regular stimulation, due mostly to the combined effect of the drop in spike frequency, and the variation in the facilitation effect. *c*, which determines fatigue, shows a similar profile between the two. (**B**) The same is plotted showing summed release over three successive 10-s periods for comparison with Figure 3 of [17]. The model shows stronger variation with the burst pattern, although a smaller drop than the data of Cazalis et al. [17] which shows a more rapid fatigue effect than the data of Bicknell et al. [14], to which our model is fitted.

The core experimental result is that phasic stimulation produces more vasopressin secretion for a given mean spike rate than regular stimulation [15]–[17]. In these experiments, isolated pituitary glands were stimulated phasically using stimulus patterns generated using recordings taken from vasopressin neurons *in vivo*. Comparing a single phasic burst and regular stimulation at the same mean frequency [17] shows a more rapid decline in the rate of secretion using the burst pattern, tested here using spike model generated data (Figure 4). The faster spiking at the head of the burst causes an increased initial rate of secretion, followed by a steep decline, due mostly to the variation in spike rate and facilitation effect, rather than increased fatigue.

**Figure 5:**
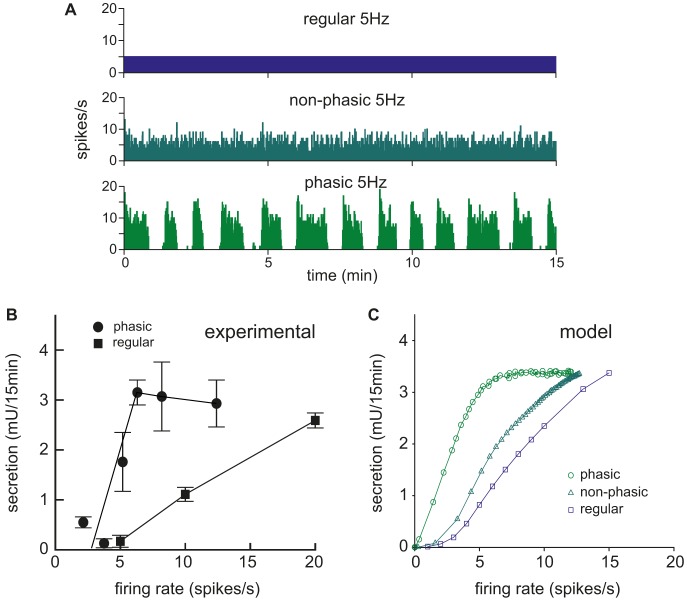
Secretion response comparing regular and phasic stimulation with increasing frequency. (**A**) Examples of regular, non-phasic and phasic model-generated spiking used to stimulate the secretion model, all with the same 5 Hz mean rate. (**B**) Data (redrawn from [15]) shows total secretion over 15-min stimulation, using regular stimulation, and stimulation triggered by recorded activity from phasic vasopressin cells. Phasic stimulation evokes much more secretion than regular stimulation at the same frequency; this is a consequence of greater facilitation of secretion at the higher intraburst firing rates, while the effects of fatigue are minimised by recovery during the silent intervals between bursts. (**C**) The model tested with a similar protocol matches the more optimal response to phasic patterned spiking, here also comparing randomly patterned non-phasic spiking. The non-phasic stimulus with periods of faster spiking at the same mean rate takes more advantage of facilitation than the regular patterned stimulus.

In [15] the secretion response to regular and burst stimulation was further tested using a range of mean frequencies, and producing the equivalent data with the model shows a close match to the *in vitro* results. In particular, for both experimental data (Figure 5A) and model data (Figure 5B), secretion is maximal in response to phasic stimulation at a mean frequency of ∼6 Hz. Similar levels of secretion can be achieved with regular stimulation, but only with much higher frequencies. In response to stimulation frequencies within the physiological range of mean firing rates for vasopressin neurons (up to ∼8 Hz), phasic stimulation always releases more vasopressin than regular stimulation. We also used the model to compare non-phasic spike patterning (by setting g*_L_* = 0 in the spiking model), simulating a continuously spiking neuron. The non-phasic pattern is more efficient than regular stimulation, but still much less efficient than the phasic pattern, as observed experimentally [16].

### Single cell model response to increasing synaptic input

**Figure 6:**
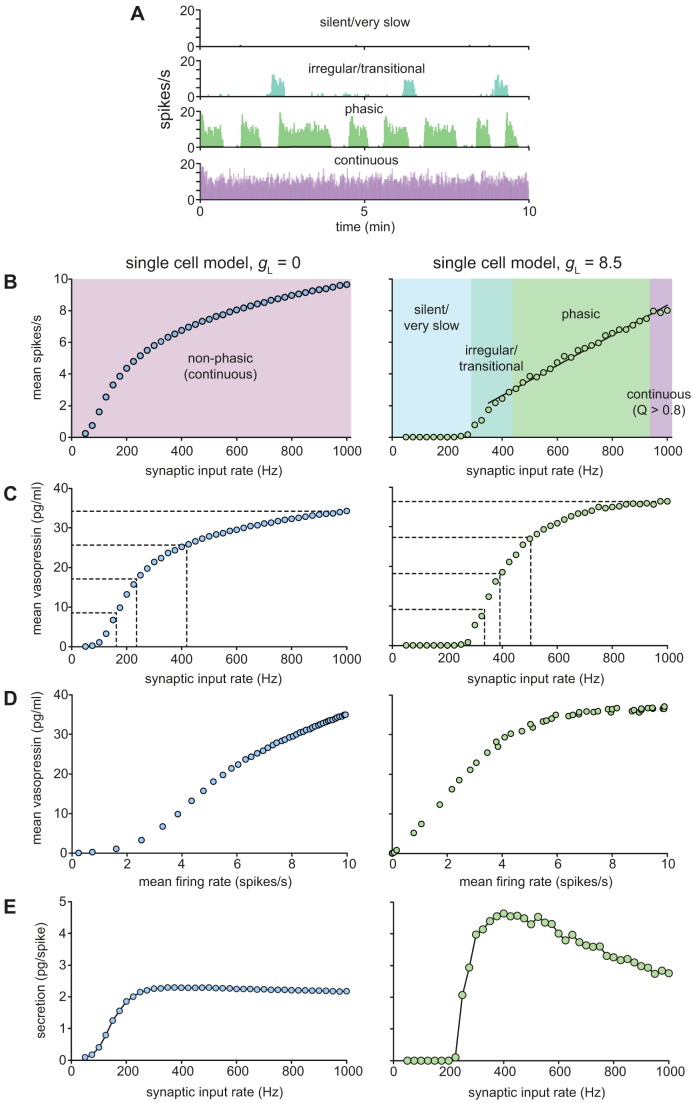
Single cell spike rate and secretion response to increasing synaptic stimulation. The spiking model (parameters in Table 1) is coupled to the fitted secretion model (Table 2). Non-phasic spiking is generated by setting g*_L_* = 0. (**A**) Examples of the four different modes of spike patterning generated by the same phasic spiking model with varied input rates. (**B**) The non-phasic model (left) shows a non-linear increase in spike rate with increased input. The phasic spiking model (right), after very little response at low input rates, shows a more linear increase in spike rates. The rate of increase is initially steep as the patterning transitions from irregular firing to full phasic spiking, but this is followed by a wide range of very linear increase as bursts lengthen and intraburst firing rate increases. (**C**) The non-phasic model (left) shows a similarly non-linear secretion response to increasing spike rate, showing a slow increase in secretion at low frequencies, followed by a facilitation driven rapid increase, which then slows along with the reduced spike rate response. The phasic secretion profile (right) shows an initial steep increase which slows as the intra-burst spike rate reaches the optimal response frequency, and longer bursts allow less recovery from fatigue. (**D**) Examining secretion against spike rate the non-phasic model shows a progressive increase in secretion with increasing frequency. By contrast the phasic model shows a very linear response up to ∼4 Hz, after which secretion becomes relatively independent of mean spike rate. (**E**) Secretion per spike shows that secretion from phasic cells is achieved with much greater efficiency at synaptic input rates exceeding 200 Hz with optimal efficiency at about 400–600 Hz, corresponding to mean firing rates of 2–4 spikes/s (comprising relatively short sparse bursts). The non-phasic cells show a less variable, but also less efficient response.

In the spiking model, we studied the effects of increasing synaptic input rate, and showed that, compared to otherwise identical non-phasic model cells, phasic cells respond to increases in input with a much more linear increase in mean spike rate. To extend this analysis to incorporate the secretory response, we coupled the spiking model to the secretion model. As previously, to investigate the adaptive consequences of the phasic firing pattern, we compared a representative phasic model cell (Figure 6A) with a cell that was identical except in lacking the mechanisms that allow the generation of phasic firing, by setting spiking model parameter *g_L_* = 0.

We simulated the plasma vasopressin concentration resulting from stimulation of the model phasic cell for 3000s at a range of fixed mean input frequencies (using 25-Hz steps from 50 Hz to 1000 Hz) by assuming a half-life of 2 min for the evoked vasopressin secretion [23]. This is a much higher rate than observed for these neurons *in vitro*, but the *in vitro* preparations are largely deafferented making this comparison unreliable; *in vivo* intracellular recordings from vasopressin neurons show a much higher rate of input than *in vitro* recordings (and a lower specific impedance) [32], but the input rates *in vivo* have not been quantified. The density of GABA synapses in the supraoptic nucleus is between 14 and 27×10^6^/mm^3^ of tissue, comprising about half of all synapses [33]. The total number of synapses per supraoptic neuron has been estimated to be ∼600 [34]; this is likely to be an underestimate, as the dendrites of magnocellular neurons extend well beyond the boundaries of the nucleus, and because it was based on an estimated neuronal population of the supraoptic nucleus that is much higher than subsequent estimates. For 600 synapses/neuron, a mean input rate of 1000 Hz represents an average input rate at each synapse of ∼1.5 Hz.

The resulting frequency-response profiles for the phasic and non-phasic cell models (parameters in Table 1) are shown in Figure 6B–E. The non-phasic model cell respond to increasing input with a non-linear increase in spike output (Figure 6B left) which is roughly mirrored by secretion (Figure 6C left). In the phasic model however, the secretion output (Figure 6C right) is much more non-linear than the spike response (Figure 6B right). Thus any possible benefits of the phasic firing pattern in linearising the cell response appear to be dissipated, and the faster firing of non-phasic cells compensates for the less efficient secretion response. Indeed, the non-phasic model appears to have a better secretion output, on the basis of dynamic range (quartiles in Figure 6C), although examining secretion against mean spike rate does show a much more linear response with phasic cells in the 0–4 Hz range (Figure 6D). The phasic cell response at faster spike rates becomes less linear, as bursts lengthen and secretion per spike becomes less efficient (Figure 6E).

### Population heterogeneity and response to increasing synaptic input

**Figure 7:**
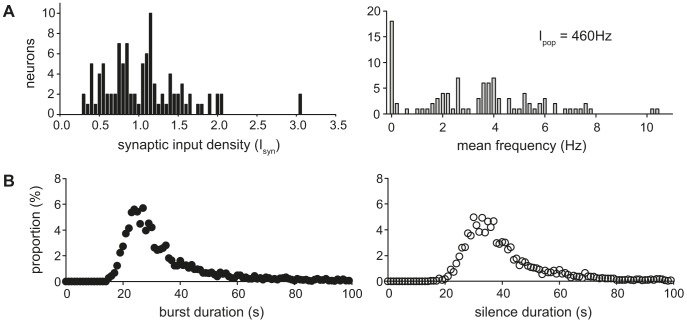
Using randomly varied synaptic input density to generate a heterogeneous 100 neuron population. (**A**) Distributions of the log normally distributed synaptic input densities (left), and the corresponding mean firing rate distribution (right), with I_pop_ = 460 Hz. The latter distribution shows a high variability in spike rates, including several almost silent cells. The phasic cells show a similar spike rate variability to experimentally recorded phasic neurons. (**B**) Distributions of burst and silence durations for the phasic population, comparable to the experimental data in [35] and [36].

One of the distinctive features of the vasopressin cell population is that it is highly heterogeneous in its spiking activity [35], [36]. During osmotic challenge, the proportion of phasically firing cells increases, but some cells still fire slowly and irregularly, while others fire more quickly in a non-phasic continuous firing pattern. Previously with the spiking model, we simulated a heterogeneous population of vasopressin cells by varying a subset of the parameters related to the phasic firing mechanism [8]. However, these variations are insufficient to reproduce the observed range of firing behaviours, and here we tested a simpler and more powerful method. Instead of varying the intrinsic properties of cells, we varied the density of synaptic input received by each cell to generate a heterogeneous population of 100 model cells, using lognormal randomly generated values. This produces a distribution of firing rates and patterns that matches closely the heterogeneity observed experimentally amongst vasopressin neurons recorded *in vivo* ([36], Figure 7A). A survey [36] of 83 recorded phasic cells showed a mean spike rate of 4.2 Hz (range 0.9 Hz to 10.7 Hz) with standard deviation 1.8. For *I*_pop_ = 460 Hz, 79 of the model population of 100 cells were fully phasic (defined as having an activity quotient [37] between 0.1 to 0.9). These phasic cells had a mean spike rate of 4.2 Hz (range 1.1 Hz to 10.4 Hz) with standard deviation 2.0, very close to the population described from *in vivo* experiments (Figure 7).

**Figure 8:**
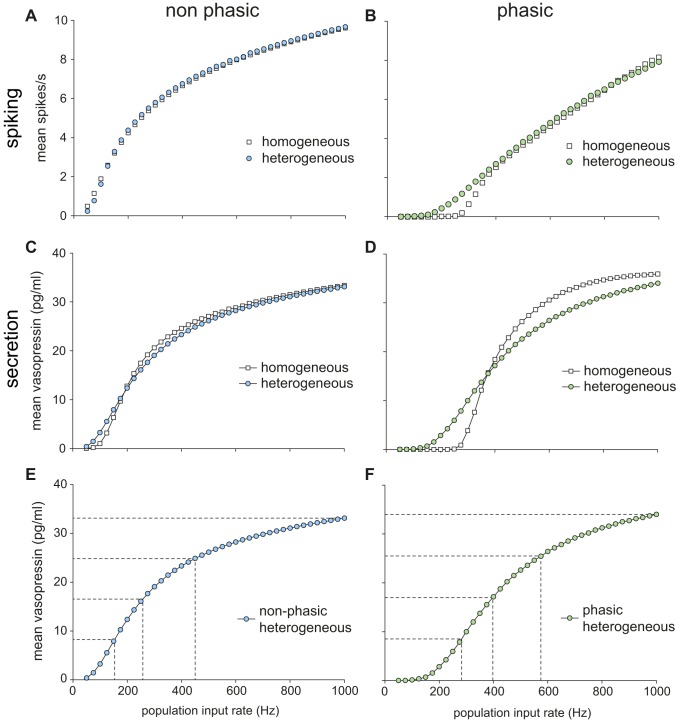
Homogeneous and heterogeneous 100 neuron population spike rate and secretion response to increasing synaptic stimulation. (**A**) Introducing heterogeneity to the population of non-phasic cells makes little difference to the mean spike rate response. (**B**) By contrast, introducing equivalent heterogeneity to the phasic cell population results in an increased linearity of the mean response to stimulation. (**C**) Introducing heterogeneity to the population of non-phasic cells produces a modest increase in the linearity of the secretory response to stimulation. (**D**) By contrast, introducing heterogeneity to the population of non-phasic cells markedly enhances the linearity of secretion. (**E, F**) Applying quartiles to the non-phasic and phasic heterogeneous populations shows a very similar dynamic range, with a slightly more linear response in the phasic cells at slower input rates.

The secretion output from these 100 cells was summed and scaled to match a full size cell population, for comparison with *in vivo* data. We compared secretion from four model populations: homogeneous and heterogeneous non-phasic cells, and homogeneous and heterogeneous phasic cells (Figure 8). The homogeneous populations comprised 100 identical cell models each with the same synaptic input density, differing only in their Poisson randomly timed synaptic input events.

Both the homogeneous and heterogeneous non-phasic populations show a similar non-linear mean spiking and summed secretion response (Figure 8A and C)) to a single non-phasic neuron (Figure 6B and C left). Similarly, the homogeneous phasic population shows just a smoothed version of the single phasic cell response (Figure 6B,C right), with a mostly linear increase in spike rate (Figure 8B) and a much more non-linear secretion response (Figure 8D). However, the input rate variation of the heterogeneous population has a strong effect on the spiking response to increased input, but only for the phasic cells. For phasic cells, heterogeneity produces a further linearization of the already more linear spike response (Figure 8B). The secretion response from non-phasic cells is modestly linearised by introducing heterogeneity – but the secretory response from phasic cells is markedly linearised and the dynamic range considerably increased.

**Figure 9:**
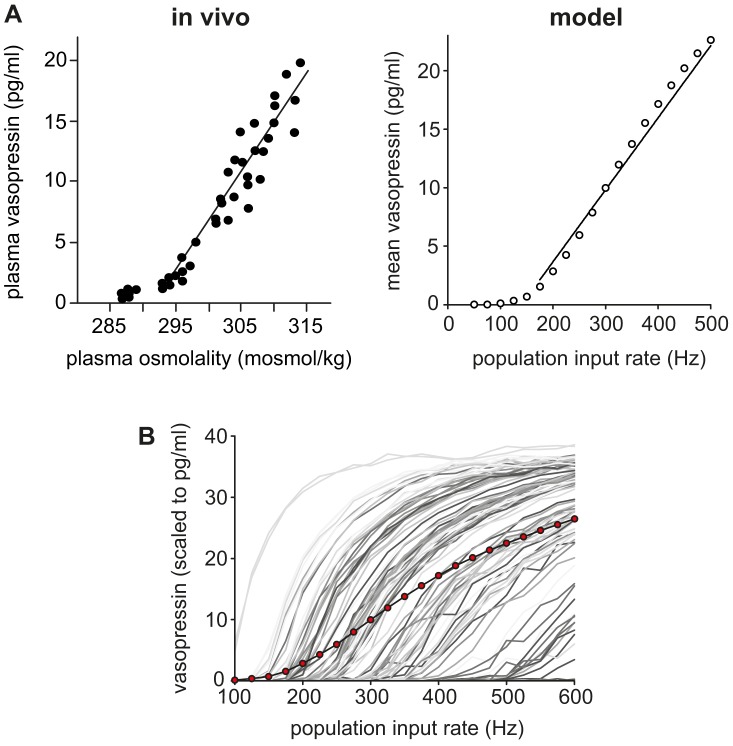
Heterogeneous phasic model cell population matched to *in vivo* secretion response. (**A**) The summed population secretion data of Figure 8D plotted on a reduced range (0 Hz to 4 Hz mean spike rate) shows a close match to the experimentally observed relationship between plasma osmotic pressure and vasopressin secretion in rats [6]. (**B**) The gray traces show the scale normalised secretion responses of the individual cells in the population. The red dots show the summed population signal. The randomly varied individual non-linear responses sum to produce a much more linear response signal.

Thus, population input heterogeneity has a strong linearising effect, but only when combined with phasic firing. Comparing the heterogeneous populations of phasic and non-phasic neurons, there is little difference in the linearity of the relationships between synaptic input and secretion (Figure 8E and F). Comparing the overall model output to the experimental data of [6] shows that the model, over the physiological range of secretion, matches the linearity of secretion in response to increased stimulation (Figure 9).

### Secretion response with long term depletion of vasopressin stores

The other challenge for the vasopressin neurons is to sustain their response during prolonged osmotic challenge, over hours and days. They must balance immediate response to osmotic challenge with preserving vasopressin stores for as long as possible, to maintain life. Depleted vasopressin stores combined with lack of access to water rapidly lead to fatal dehydration.

**Figure 10:**
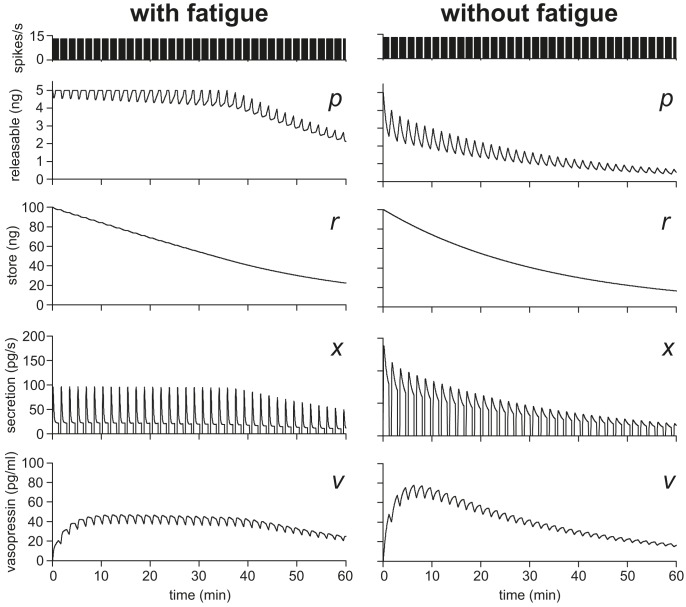
Single cell secretion during prolonged stimulation. The reserve store capacity was set to a reduced 0.1 µg (*r*_max_ = 100000) in order to observe depletion on an accelerated timescale. Stimulation was 72-s bursts of regular 13 Hz spiking with 30-s silences, sustained for 60 min. We compared the model with and without fatigue (*c*_inhib_ = 1). With fatigue, secretion is limited to a rate where the silent period is sufficient to restore the releasable pool and maintain the secretion response, until reserve store is ∼50% depleted. Without fatigue, bursts initially trigger a much higher secretion rate and the releasable pool cannot be maintained, so that the secretion rate is directly proportional to the declining reserve store.

Vesicle stores are represented in the model as a reserve store (*r*) and a releasable pool (*p*). The normal vasopressin content of the rat pituitary is approximately 1 µg [23]. However, to study the effects of depletion in the model on a timescale where we can still observe the response to individual spike bursts, we set the initial content to a very low value (*r*_max_ = 100000, equivalent to a content of 0.1 µg) and simulated stimulation in 72-s bursts separated by 30-s silences, with regular intraburst activity at 13 Hz (Figure 10). The content of *p* (*p*_max_ = 5000), which is much smaller than the reserve store, was set large enough to support secretion during a typical burst with only partial depletion (Figure 10) but also small enough to be fully replenished during a 30-s silence. We compared the model with and without fatigue, removing the effect of *c* by setting *c_inhib_* = 1. In the model, *p* is replenished at a rate proportional to *r*. While this rate is greater than *x* (the rate of secretion) *p* is maintained near its maximum, and the relation between spike rate and secretion is maintained. Only when *r* is sufficiently depleted (∼50%) does the secretion response decline. Thus, it seems that fatigue helps to maintain a consistent response to stimulation in the face of progressive store depletion.

**Figure 11:**
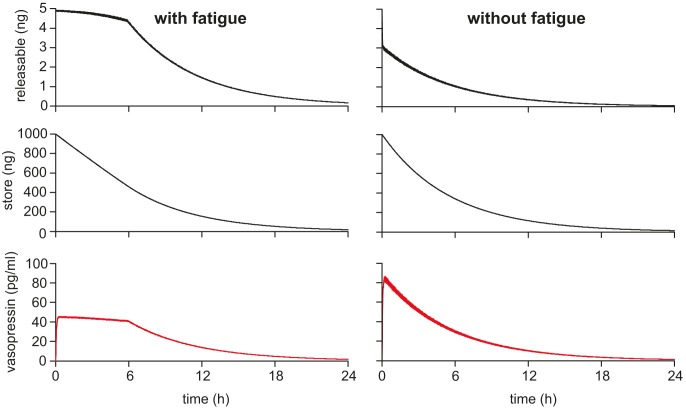
Single cell secretion during long term store depletion. The reserve store capacity was set to 1 µg (*r*_max_ = 1000000) to match the normal pituitary content. Stimulation used the spiking model with synaptic input rate *I*_re_ = 600 Hz, and was sustained for 24 h. As in Figure 10, we compared the model with and without fatigue (*c*_inhib_ = 1). The results are similar to Figure 10 in that, with fatigue, secretion is maintained at a constant level for about 6 h despite progressive depletion of the store; only when the reserve store is ∼50% depleted does secretion become proportional to store content. Without fatigue, secretion is proportional to store content throughout.

We then tested this with a simulation setting *s*_max_ = 1000000 (1 µg) and using spike model generated activity instead of regular stimulation. The spike model input rate was set at 600 Hz, producing a mean spike rate of 4.7 Hz (at the high end of the physiological range). Figure 11 shows the model output, with and without fatigue, for 24 h of simulated stimulation. With fatigue, secretion is maintained at a fairly constant mean level for about 6 h, before declining progressively. Without fatigue, secretion declines from the beginning. Thus, in the model, the presence of a fatigue mechanism enables a consistent response to stimulation to be maintained until the stores have reached about 50% depletion.

### Secretion response during long term depletion in a heterogeneous population

**Figure 12:**
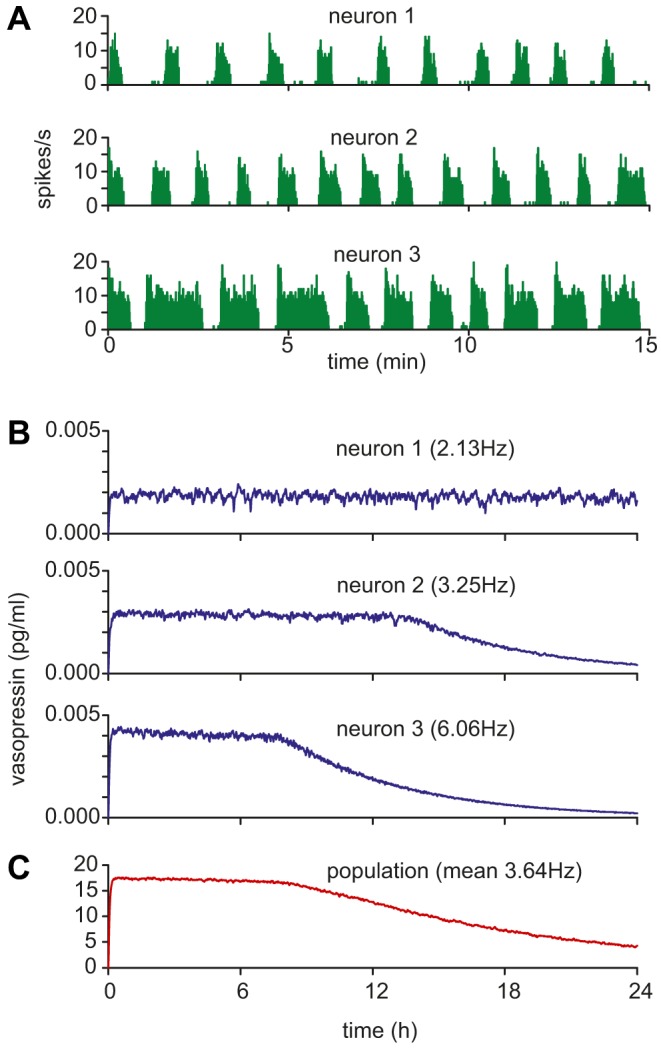
Secretion during store depletion in a heterogeneous population. We used the same 100 neuron heterogeneous population as in Figures 8 and 9 with *r*_max_ = 1000000 and simulated 24 h of activity with *I*_pop_ = 400 Hz, equivalent to osmotic stimulus during sustained dehydration. The mean spike rate in the population was 3.64 Hz. (**A**) The spiking activity in three typical phasic patterned neurons from the population (neuron 1, *I*_syn_ = 0.9302; neuron 2, *I*_syn_ = 1.1630; neuron 3, *I*_syn_ = 1.9023) showing varied rates of spike activity, including varied burst and silence durations. (B) The corresponding single cell vasopressin concentrations (scaled to approximate single cells *in vivo*, assuming a 9000 cell population). The rate of depletion, and the period for which the cell sustains its secretion rate varies with mean firing rate. The slowest cell is able to sustain secretion for the full period. (**C**) The summed population output declines from a time which depends on the fastest depleting cells in the population, but the decline in secretion is slower and more linear than the store-dependent decline observed in single cells.

In a heterogeneous population, individual cells will become depleted at different times. To study this, we used the same heterogeneous population as used in Figures 8 and 9, and simulated stimulation for 24 h with a population input rate of 400 Hz (Figure 12A). The slowest cells maintain their response for the whole 24 h, but most active cells become depleted, and their responses become proportional to their respective reserve store levels well within 24 h (Figure 12B). However, although the decline in the overall secretion of the population (Figure 12C) begins at a time determined by the most active cells, heterogeneity in the input rates results in heterogeneity in depletion rates, which reduces the rate of the decline in the population signal. We know from experimental data [38] that, when tested over up to 4 days of stimulation, vasopressin secretion declines in proportion to pituitary content, but there is currently not enough data to make a more detailed quantitative comparison with the model.

## Discussion

The core aim of this study was to understand how the properties of the secretion mechanism in vasopressin neurons relate to their signal processing abilities and function. We developed a differential equation based model of the secretion mechanism, based on concise representations of the underlying mechanisms, and this model reproduces the non-linear facilitation and fatigue effects observed experimentally. By coupling this secretion model to an integrate-and-fire based spiking model, we created a full cell model which we used to investigate the relationship between synaptic input activity and secretion response, for both single cells and a neuronal population.

We are aware of only one previous published model of the secretion response in vasopressin neurons [39]. This used an *ad hoc* representation, based simply on a piecewise linear fit to experimental data [12], and it does not produce a response which is sensitive to spike patterning (differentiating phasic from regular stimulation), which is one of the key features of vasopressin cells.

### Ca^2+^ based model mechanisms

The present secretion model implements mechanistic elements derived from previous experimental studies. The rate of vesicle exocytosis depends on Ca^2+^ entry, and the model relates Ca^2+^ entry at the axonal terminals to spike activity. Facilitation of stimulus-secretion coupling arises as a consequence of the spike broadening that has been observed in recordings from the terminals. We assumed that most of the increase in spike duration reflects a high voltage-gated Ca^2+^ conductance, since the effect is blocked when Ca^2+^ is removed [9]. This results in a frequency-dependent increase in Ca^2+^ entry per spike. In vasopressin cells facilitation of stimulus-secretion coupling peaks at ∼15 Hz. By contrast, oxytocin cells continue to show increasing facilitation at spike rates as high as 50 Hz, and this suggests that vasopressin cells have some other competing inhibitory mechanism. To fit the measured frequency-response profile [12], we added a Ca^2+^-dependent inactivation of Ca^2+^ entry.

We modelled fatigue of stimulus-secretion coupling as the effect of a Ca^2+^-activated K^+^ current that reduces the probability that axonal spikes invade the terminals. The close match between intracellular Ca^2+^ time course and the development and recovery from fatigue [14], [17], the existence of suitable Ca^2+^ activated K^+^ channels, and activity-dependent spike failure [27], all make this mechanism plausible, and we chose model parameters to match experimental data quantitatively. The data of [17] suggests a more rapid development of fatigue than [14]. The model is capable of fitting both, but for the results here we use parameters that fit our own data [14]. In both cases, the time resolution is limited, and ideally we would repeat the experiments, using spiking model generated data. We also experimented with an extended mechanism incorporating ATP- and adenosine-based mechanisms [40], that are thought to modulate the Ca^2+^ activated K+ channel and an N-type Ca^2+^ channel respectively, but the improvement in fit was not sufficient to justify the increased complexity.

### Vesicle stores and depletion

Although the model doesn't use a releasable store to simulate fatigue, it does use a store mechanism to simulate the depletion that occurs on longer timescales, with the model tested for durations up to 24 h. The secretion of vesicles from the posterior pituitary has been studied quantitatively at the ultrastructural level [41]; these studies led to the conclusion that exocytosis can occur from all parts of the axons of magnocellular neurons – from nerve terminals, from the large axonal swellings that are a conspicuous feature of the gland and which contain most of the vesicles, and even from undilated axons. However in each of these compartments, and under different physiological conditions including different states of depletion, the number of vesicles that is released by a given stimulus appears to depend only on the number of vesicles that are close to the plasma membrane [41]. This implies that in any secretory compartment, exocytosis is a stochastic process, and that the probability of release from a compartment depends on the content of a releasable pool in that compartment. On long timescales (tested over 4 days) the rate of secretion is proportional to the total gland content [38], suggesting that replenishment of the releasable pool (close to the plasma membrane) from a deeper store is also activity-dependent and probabilistic.

In normal conditions the system functions to maintain body fluid homeostasis, and secretion is linearly proportional to osmotic pressure. We assume that it is also important to maintain a consistent response, and therefore an advantage to make secretion independent of the store content. However, in conditions of prolonged challenge it will be more important to preserve vasopressin stores, so that osmotic pressure can be maintained at a life preserving level for as long as possible, and so it would be of use to gradually reduce the secretion rate, as rationing becomes more urgent.

In modelling the store and the releasable pool, we assumed that secretion is proportional to the releasable pool, and that it is refilled at a rate proportional to the store content. We did not include any synthesis in the model, as the long axons mean that there is a long delay (24 h) between an increase in demand and an increase in the arrival rate of newly synthesised vesicles at the terminals [42]. In the present model, the releasable pool represents the summed total of all the terminals of a single cell. It was set large enough that a single burst would release only a small proportion of the content, with a refill rate parameter fast enough to refill the pool between bursts while the store content is at maximum. Under these conditions, we get a model cell that, in response to a large and sustained increase in activity (comparable to that which accompanies systemic dehydration) can maintain a stable secretion rate for a long period (hours). However, once the store reaches a critical level of depletion, the releasable pool also becomes depleted, and thereafter the secretion rate becomes proportional to store content.

These simulations give a strong indication of the likely adaptive value of fatigue. From experimental data we know that fatigue of stimulus-secretion coupling is a marked characteristic of vasopressin secretion but not of oxytocin secretion – so it is not an inevitable feature of stimulus-secretion coupling in neuroendocrine neurons. The present model results suggest that in conditions of chronically maintained stimulation, fatigue is important for maintaining a constant level of output despite declining stores. This may be important physiologically for vasopressin secretion, where the absolute level of vasopressin determines the degree of antidiuresis, but less important for oxytocin in its primary role as a hormone that mediates milk let-down – a reflex governed not by the mean sustained level of oxytocin secretion but by the frequency at which transient pulses of secretion occur [43].

### Single cell secretion response

Combining the new secretion model with our previously published spiking model allows us to examine the relationships between synaptic input, spiking activity, and secretion. As we showed previously, because the phasic activity of vasopressin cells is asynchronous, and because of the properties of the mechanism that generates phasic activity, the average spike rate of the population increases relatively linearly in response to increased afferent input despite the short term non-linearity of individual cell responses. However, as we show here, the combined effects of fatigue and facilitation make the secretory response to increasing input one that is highly non-linear. This is an obvious consequence of facilitation, which increases secretion per spike as spike rate increases. However, fatigue also makes the response less linear, by capping the secretion rate for a proportion of the spike activity - a proportion that increases as burst durations increase. The effect of this overall nonlinearity is to reduce the dynamic range of the secretory response compared to that of the spiking response.

### Population response and heterogeneity

However, to properly understanding the relationship between the underlying mechanisms and the function of the cells, we need to look not at individual cells, but at how they behave as a population. To do this we used populations of 100 model cells to look at the relation between synaptic input (assumed to be representative of osmotic pressure) and summed population secretion, scaled to compare against full size cell populations (∼9000 cells).

Testing a homogeneous population of 100 identical model cells has the expected smoothing effect of an averaged population signal, but otherwise matches the features of the single cell response. However, vasopressin neurons are a highly heterogeneous population, varying both in their firing rates, and spike patterning. Previously, with the spiking model, we simulated heterogeneity by adding random variation to a subset of the parameters which had been used to fit varied recorded cells. This is sufficient to produce a range of phasic firing patterns, but not the highly varied activity that is observed *in vivo*, and this is likely to underestimate population heterogeneity since identified cells are biased towards the more distinctive phasic pattern.

To produce more varied spike rates we used a simpler method, applying random variation to the rates of synaptic input received by each model cell, using a log normal distribution (Figure 8A). Tests using a heterogeneous population using the subset method showed only minor variation from the homogeneous response profile (not shown). However, tests with input based heterogeneity show a marked linearisation of the population response, along with a large increase in the dynamic range, producing a profile which is much closer to the highly linear secretion response observed *in vivo* (Figure 9). The effect only works using the phasic cell model, with non-phasic cells showing very little difference between homogeneous and heterogeneous populations.

### The adaptive value of heterogeneity

The vasopressin neurons of the supraoptic nucleus are probably as homogeneous a population of neurons as any that exists within the CNS: there are about 2000 of these in each nucleus, and each has just a single axon that projects to the posterior pituitary gland with few if any collateral branches that terminate in the CNS. Thus they share a strictly common function in that the role of each is to contribute to the pooled output of vasopressin that enters the systemic circulation. While these neurons have many features in common, there is also considerable variability in their intrinsic properties, and their spontaneous firing rates and patterns vary substantially from cell to cell – even between immediately adjacent cells recorded simultaneously. The most parsimonious explanation of this variability is that it is not “designed in”, but is the consequence of intrinsic variability in the developmental determination of gene expression and neuronal wiring patterns. But however this variability arose, the question arises of whether it has any adaptive significance: does this variability affect information coding, and does it do so in a way that yields any apparent advantages?

This question has been addressed previously for several other neuronal systems, leading to the conclusion that there can indeed be various advantages of neural heterogeneity in information coding [44]–[46]. However, these previous studies all drew their conclusions from models that assumed that information is coded in the spike activity of neurons. However the information that is transmitted from neuron to neuron is mediated not by spike activity itself, but by the secretion that is evoked by that activity – and in all neurons the coupling between activity and neurosecretion is complex and highly nonlinear. Here we show that in the case of vasopressin cells at least, taking account of stimulus-secretion coupling radically changes our perception of the importance of heterogeneity.

Here, we have modelled closely the particular properties of stimulus-secretion coupling in vasopressin cells. These properties vary in different neuronal types, but it seems that frequency facilitation is a common if not universal feature of neurotransmitter secretion from synapses, and that activity-dependent depletion and other mechanisms functionally equivalent to fatigue are also common if not universal. We have shown here that the functional properties of a network critically depend upon these properties. The output of a neuron is not its spike activity, but what it secretes in response to that activity, and representing this accurately in network models may be critical to properly understanding how they process information.
